# Microfluidic biosensor for β-Hydroxybutyrate (βHBA) determination of subclinical ketosis diagnosis

**DOI:** 10.1186/s12951-015-0076-6

**Published:** 2015-02-12

**Authors:** Xuan Weng, Wenting Zhao, Suresh Neethirajan, Todd Duffield

**Affiliations:** BioNano Laboratory, School of Engineering, University of Guelph, Guelph, ON N1G 2W1 Canada; Department of Industrial Engineering, South China University of Technology, Guangzhou, Guangdong 510640 China; Department of Population Medicine, Ontario Veterinary College, University of Guelph, Guelph, ON N1G 2W1 Canada

**Keywords:** β-hydroxybutyrate (βHBA), Microfluidic biosensor, On-site diagnostics, Subclinical ketosis

## Abstract

**Background:**

Determination of β-hydroxybutyrate (βHBA) is a gold standard for diagnosis of Subclinical Ketosis (SCK), a common disease in dairy cows that causes significant economic loss. Early detection of SCK can help reduce the risk of the disease progressing into clinical stage, thus minimizing economic losses on dairy cattle. Conventional laboratory methods are time consuming and labor-intensive, requiring expensive and bulky equipment. Development of portable and robust devices for rapid on-site SCK diagnosis is an effective way to prevent and control ketosis and can significantly aid in the management of dairy animal health. Microfluidic technology provides a rapid, cost-effective way to develop handheld devices for on-farm detection of sub-clinical ketosis. In this study, a highly sensitive microfluidics-based biosensor for on-site SCK diagnosis has been developed.

**Results:**

A rapid, low-cost microfluidic biosensor with high sensitivity and specificity was developed for SCK diagnosis. Determination of βHBA was employed as the indicator in the diagnosis of SCK. On-chip detection using miniaturized and cost-effective optical sensor can be finished in 1 minute with a detection limit of 0.05 mM concentration. Developed microfluidic biosensor was successfully tested with the serum samples from dairy cows affected by SCK. The results of the developed biosensor agreed well with two other laboratory methods. The biosensor was characterized by high sensitivity and specificity towards βHBA with a detection limit of 0.05 mM.

**Conclusions:**

The developed microfluidic biosensor provides a promising prototype for a cost-effective handheld meter for on-site SCK diagnosis. By using microfluidic method, the detection time is significantly decreased compared to other laboratory methods. Here, we demonstrate a field-deployable device to precisely identify and measure subclinical ketosis by specific labeling and quantification of β-hydroxybutyate in cow blood samples. A real-time on-site detection system will maximize convenience for the farmers.

## Background

Subclinical ketosis (SCK) is characterized by the increase in the concentration of circulating ketone bodies without the presence of clinical signs of ketosis [1]. SCK is a common disease in high-producing dairy cows and typically occurs in early lactation [2]. It is reported that the incidence of SCK may be as high as 40 ~ 60% herds, which is much higher than the 2 ~ 15% incidence of clinical ketosis [2,3]. SCK has been found to be associated with decreased milk production, impaired reproductive performance, displaced abomasum and higher risk of clinical ketosis, thus causing huge economic losses [4,5]. To prevent SCK from becoming a clinical disease and to minimize economic losses, it must be identified at an early stage for cows so that effective treatment can begin. However, it is difficult to identify SCK due to the lack of clinical signs and it may be undetected by regular clinical ketosis tests.

β-hydroxybutyrate (βHBA) is considered a gold standard for diagnosing of SCK due to its stability in blood. A cut-off value of 1.2 to 1.4 mM of βHBA in blood samples is used to distinguish between cows with and without SCK [6-8]. Conventional quantitative determination for βHBA is conducted in a laboratory by using special equipment and requires time-consuming, labor-intensive procedures and skilled personnel [9,10]. Current available commercially kits for βHBA determination (Cayman Chemical® β-Hydroxybutyrate (Ketone Body) Colorimetric Assay Kit; Abcam’s beta Hydroxybutyrate (beta HB) Assay; BHBA ELISA Kit from antibodies-online Cow (Bovine); typically based upon an enzymatic catalysis followed by fluorescence, absorbance or electrochemical detection which still involve complex procedures and depend on special and expensive optical instrument for signal detection. Usually, a relatively high sample volume and reagent amount is used, resulting in a lengthy assay time. Although the methods achieve high sensitivity and specificity, all of the aforementioned limitations prevent field deployable and on-site applications.

Recently, numerous “cowside” tests for diagnosis of ketosis based on βHBA determination have been developed. Although some of the cowside diagnostic tests have been commercially available for ketosis by detecting βHBA (e.g., Ketolac, Biolab, München, Germany), they can only provide semi-quantitative results [11,12]. More recently, human ketosis detector has been used as cowside ketosis test [7,13-18]. Evaluation of human ketosis handheld devices in the determination of βHBA in cow’s blood samples provided moderate to excellent levels of agreement with the laboratory tests. However, the results of the evaluation of Optimum Xceed human ketosis hand-held meter by Voyvoda and Erdogan [12] showed that the sensitivity for βHBA detection in comparison with laboratory test was less than 85%. Furthermore, since these tests are designed specifically for use on humans, veterinary application may not be supported and the use of these tests could be lost if the manufacturer decides the human market does not support sales. Hence, it is clear that the human ketosis detector may not be applicable for consistently accurate and reliable measurement of βHBA in cow’s blood and animal samples. The study by Mahrt et al. [18] states that the Novavet biosensor provided only 82% specificity in βHBA in comparison to the lab tests, with significant false-positive results indicating that this biosensor may not be suitable for on-farm detection of sub-clinical ketosis.

Therefore, motivated by the limitations of current devices for cowside SCK diagnosis, we have developed a high performance microfluidic biosensor to meet the market needs. The uniqueness of the presented biosensor is that it is based on a microfluidic system with superior specificity and sensitivity along with the higher accuracy for determination of βHBA in serum samples of cows. Microfluidics and lab-on-a-chip technology have been widely used in biosensor applications, enhancing analytical performance by miniaturization. Microfluidic biosensors present distinctive advantages such as significantly increasing sensitivity with reduced assay time and reduced sample and reagent consumption [19]. In order to make a cost-effective biosensor, expensive, bulky and complex optical instruments such as FTIR, (NMR) spectroscopy spectrometry and gas chromatography-mass spectrometry (GC-MS) have to be avoided. The objective of this study was to develop a low-cost, highly sensitive and miniaturized microfluidic biosensor as a handheld device capable of rapid, accurate detection of βHBA for SCK diagnosis in dairy cows.

## Materials and methods

### Reagents and materials

The reagents and materials were obtained as a commercial kit (Cayman Chemical® β-Hydroxybutyrate (ketone Body) Colorimetric Assay kit, Cedarlane labs, Burlington, ON, Canada) which consisted of βHBA assay buffer, reconstitute βHBA standard, βHBA enzyme solution and βHBA colorimetric detector. The βHBA standard solution ranging from 0.05 mM to 1.0 mM was made by diluting the reconstituted βHBA Standard with βHBA assay buffer to prepare the βHBA standard curve. The βHBA enzyme solution and colorimetric detector were mixed to make the detector mixture buffer at a ratio of 24:1 before each test.

### Preparation of serum sample

Blood samples collected from periparturient Holstein dairy cows from 4 dairy farms in South West Ontario were utilized for analysis. The detailed sample preparation procedure can be found elsewhere [20]. Briefly, cow’s blood was first collected from the coccygeal blood vessels in a vacuum tube without anticoagulant and stored in a cool place. Collected blood sample was centrifuged at 4°C within 6 h after collection at 2990 × g to harvest serum and stored at -20°C for further use. Before assaying, the serum sample was thawed at room temperature and diluted 1:6 with the βHBA assay buffer.

### Principle of the colorimetric assay

**Figure 1:**
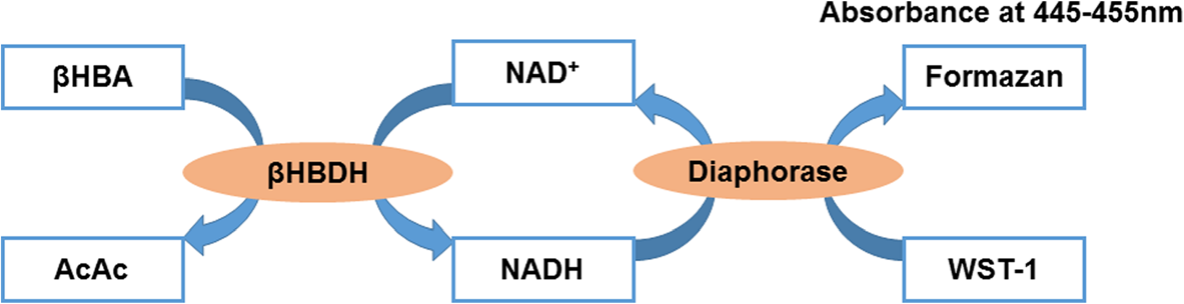
**Principle of enzymatic βHBA formation used in this study.**

The method for βHBA determination in this study is based upon the enzymatic conversion of βHBA to acetoacetate (AcAc) by βHBA dehydrogenase, and concomitantly the cofactor nicotineamide adenine dinucleotide (NAD^+^) is converted to its reduced form β-nicotinamide adenine dinucleotide (NADH). In the presence of diaphorase, NADH reacts with the colorimetric detector WST-1 to produce a formazan dye with a maximum absorbance at 445 nm to 455 nm, proportional to the βHBA concentration. Figure 1 shows the principle of βHBA determination based upon the enzymatic reaction.

### Design and fabrication of microfluidic PDMS chip

**Figure 2:**
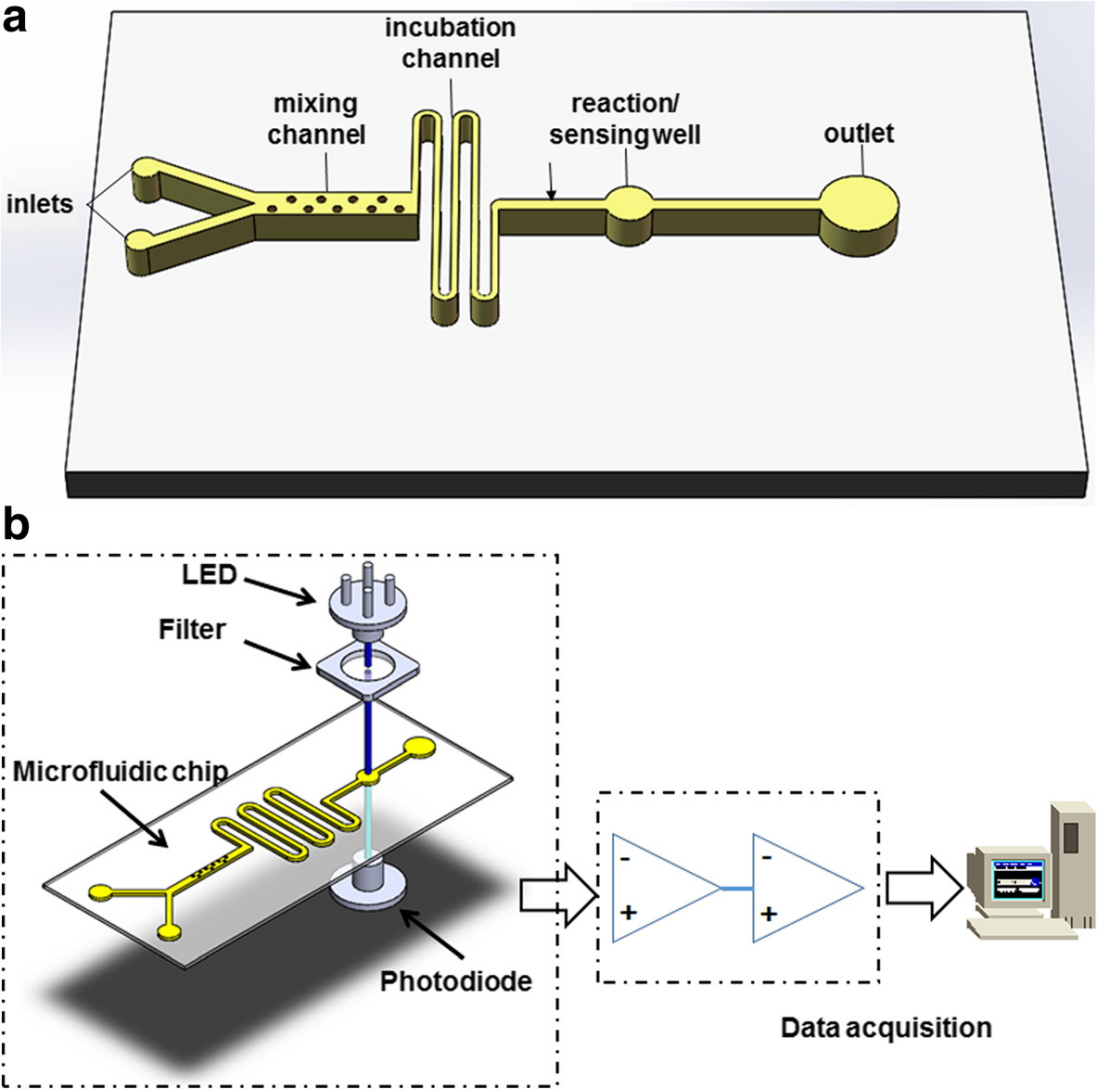
**Microfluidic chip design. (a)**: Schematic diagram. The microfluidic chip had one outlet and two inlets for βHBA standard solution or serum sample and the detector mixture, respectively. The main channel of 200 μm wide and 60 μm deep consisted of a mixing channel, incubation channel and a sensing well. Hundreds of posts arranged in a zigzag line were designed in the mixing channel to enhance the mixing effect. A long pathway for liquid was designed for further mixing enhancement and incubation. The diameter of the sensing well was 2.5 mm, which was comparable to the sensing area of the Si photodiode. The flow was driven by the capillary forces. **(b)** The picture of the microfluidic chip filled with a blue dye for visualization.

The microfluidic chip design was created using AutoCAD software and made by following the standard soft lithography protocol as detailed by Biddiss et al. [21]. The layout of the microfluidic chip is shown in Figure 2a. The microfluidic chip consists of the mixing channel housing hundreds of posts, incubation channel, sensing well, two inlets and one outlet. A silicon wafer master mold was fabricated by first spin-coating a thin layer of SU-8 2025 negative photoresist (MicroCHEM, US) on the surface of the wafer. After the prebaking, a photomask with the designed microchannel geometry was placed onto the coated silicon wafer and exposed to UV using a UV exposure system (UV-KUB, Kloé, France). After the post-baking and developing, it was used as a mold for creating the PDMS chip. A 10:1 (w/w) mixture of PDMS prepolymer and the curing agent (Sylgard, Dow Corning, Burlington, ON, Canada) was stirred thoroughly and degassed under vacuum. Then the mixture was poured onto the master mold and cured at 75°C for 4 h. After curing, the PDMS replica was peeled off from the master, punched with holes to provide inlets and outlet and bonded onto a glass slide (25 × 75 × 1 mm, VWR International, Suwanee, GA, USA) after oxygen plasma treatment for 40 s. A position marker was used to facilitate the alignment between the sensing well and the sensing window of the photodiode mounted in a custom design box when conducting the bonding. A picture of microfluidic chip is shown in Figure 2b.

### Miniaturized, low-cost optical biosensor

The principle of the biosensor is based upon the property of the resulting complex to absorb UV in the 445 to 455 nm range. The transmitted light intensity signal acquired by the Si photodiode is dependent on the βHBA concentration in the samples.

**Figure 3:**
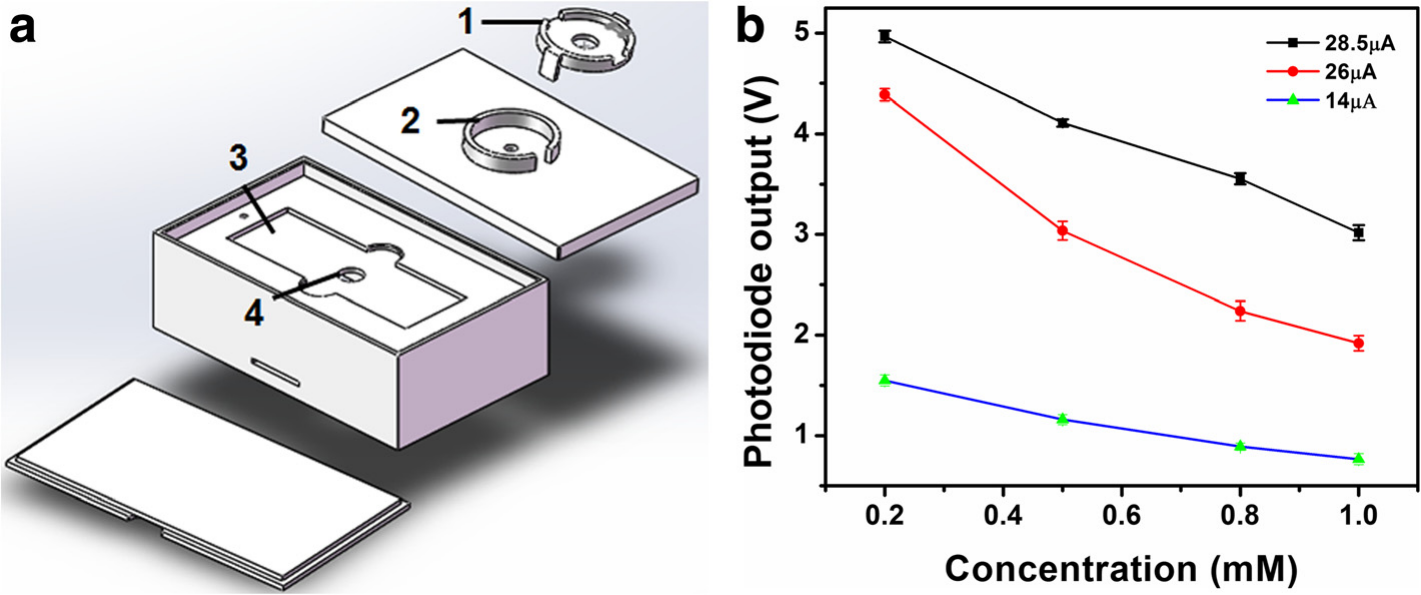
**Schematic diagram of the optical biosensor. (a)** Components of the optical biosensor. **(b)** Custom-built biosensor packaging for optical and electrical components assembly. Labels 1, 2, 3, 4 indicate the positions for mounting LED, bandpass filter, microfluidic chip and Si photodiode, respectively.

The light absorption analysis was performed by a custom-built miniaturized and low-cost optical biosensor. The schematic diagram of the optical sensor is shown in Figure 3a. The biosensor used a LED (447.5 nm, Luxeon Rebel, Luxeon Star LEDs, Brantford, ON, Canada) with a single-band bandpass filter (435 nm, semrock, Rochester, NY, USA) as the illumination light source and a low-noise, high-sensitivity Si photodiode (Hamamatsu, Bridgewater, NJ, USA) with preamp as the detector for light absorption analysis. The collected light intensity signal by the Si photodiode was then digitized and transferred to a PC for storage by a programmable microcontroller (Arduino Uno, SparkFun Electronics, Niwot, CO, USA) through an interface circuit. Optical and electrical components were assembled in a sensor packaging assembly to block ambient light, as shown in Figure 3b. The LED and filter were mounted in the lid and on the top of the microfluidic chip platform to illuminate the sensing micro-well. The Si photodiode was placed at the bottom of the platform aligned to the sensing well.

### Detection procedure

As mentioned previously, βHBA determination was based upon an enzymatic reaction. First, a 1:1 ratio volume of βHBA standard solution or diluted serum sample and the detector mixture containing βHBA enzyme solution and βHBA colorimetric detector were loaded into the respective inlets of the microfluidic chip placed on the chip platform. The two solutions underwent full mixing in the mixing channel and then passed through the incubation channel and the sensing well to the outlet. After the incubation time of 1 min, the power supply and the Arduino microcontroller were activated for signal recording. All experiments were conducted at room temperature.

**Figure 4:**
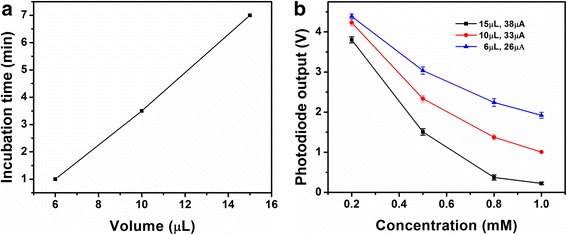
**Determination of incubation time. (a)** Response of the biosensor at various time points for a βHBA standard solution of 0.5 mM at the mixture volume of 10 μL with LED input current at 33 μA. **(b)** Relationship of the saturation incubation time and mixture volumes.

**Figure 5:**
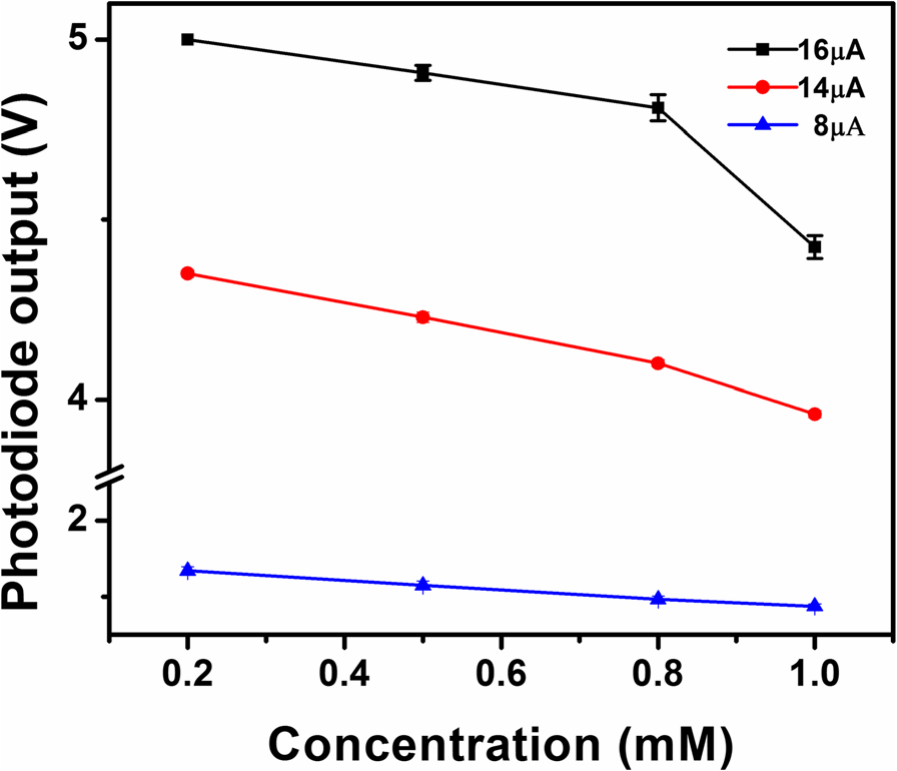
**βHBA standard curve of biosensor by different volume with respective optimized light intensity.**

**Figure 6:**
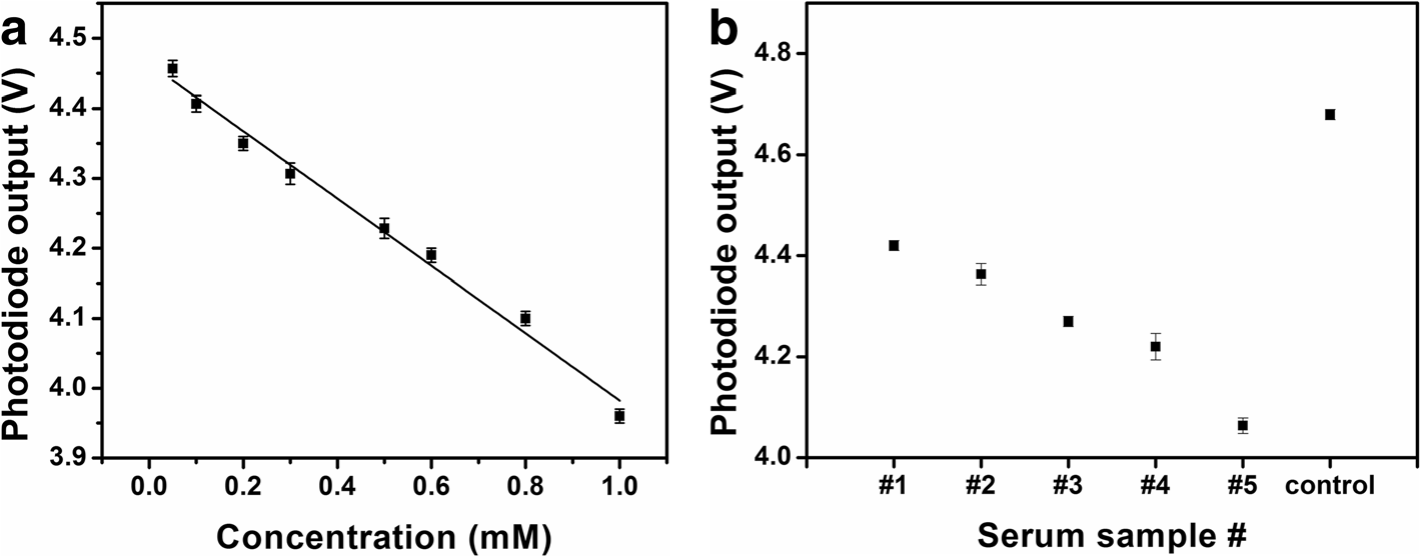
**Response of biosensor for the on-chip test at a volume of 0.2 μL. (a)** Determination of LED light intensity by detecting different concentration of βHBA standard solution. **(b)** βHBA standard curve of the microfluidic biosensor.

The light signal was recorded for 5 s. For each experiment, the standard deviation (SD) for signals from 3 duplicate tests was calculated, and is indicated by the error bars (Figures 4, 5, and 6). The detection limit, sensitivity and specificity of the biosensor were evaluated based on the experiments.

The βHBA concentration of the serum sample was detected by three different approaches to evaluate the performance of the presented biosensor. Same samples of serum were analyzed using a Roche Cobas 6000 c501 automated chemistry analyzer (Roche Canada, Laval, QC, Canada), a Synergy H4 Hybrid Multi-Mode Microplate Reader (Biotek, Winooski, VT, USA) and the developed microfluidic Lab-on-a-Chip biosensor, respectively. The values of βHBA in a sample were calculated using the linear regression equations of their own standard curves obtained by each approach.

### Determination of the operating parameters

The principle of the biosensor is based upon light absorption analysis. The LED served as the illumination light source to the biosensor. The transmitted light intensity signal is dependent on the βHBA concentration, was captured by a Si photodiode. It was found that the parameters of applied LED intensity, the volume of the resultant complex and the incubation time were interconnected, and optimization of these parameters was performed in order to obtain a stable, effective and reliable outcome with high sensitivity. A series of experiments using standard βHBA solution ranging from 0 ~ 1.0 mM were performed to study and optimize the operating parameters. A different light intensity from LED was obtained by adjusting the voltage applied on LED. A series of volumes of resultant complex associated with the optimized incubation time for each one were studied. Well-based tests with relatively big volumes (15 μL, 10 μL and 6 μL) of reaction mixture were first evaluated to investigate the feasibility and performance of the biosensor. Based on the initial optimization experiments, on-chip tests using the microfluidic chip were then conducted.

## Results and discussion

### Optimization of operating parameters based on well-based tests

**Figure 7:**
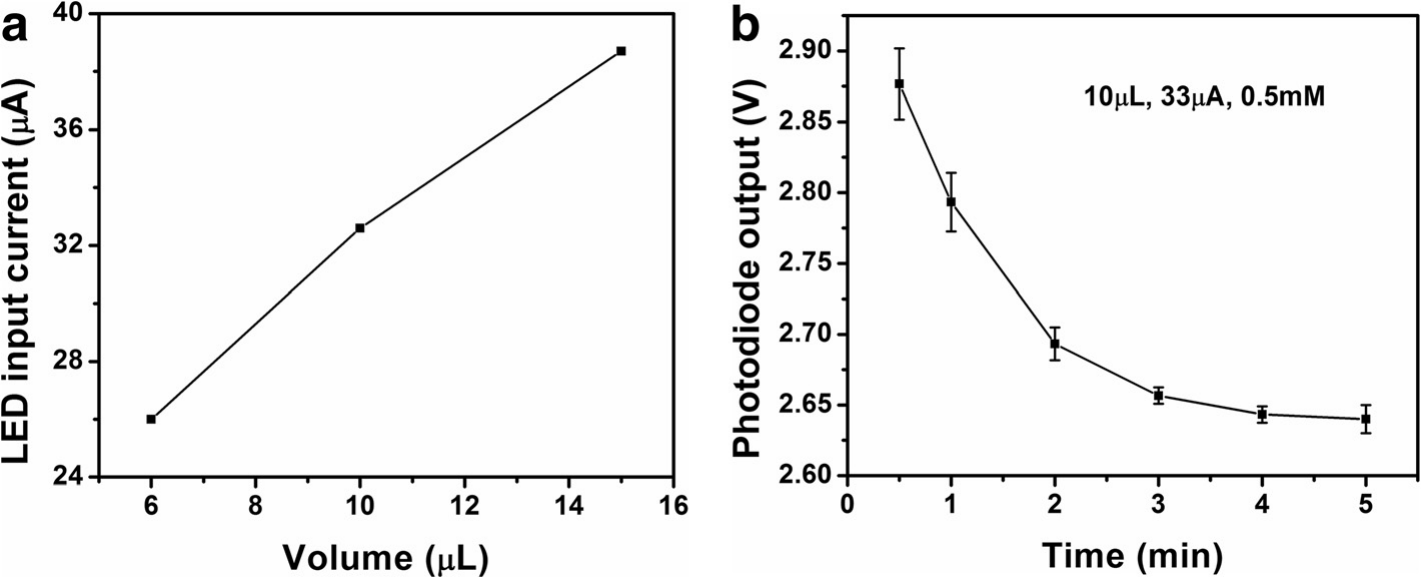
**Determination of optimized LED light intensity. (a)** Response of biosensor with different LED light intensity for different concentration of βHBA standard solution at a volume of 6 μL. **(b)** Optimized LED light intensity for different volumes.

Three operating parameters: illumination light intensity, sample/reagent volume and incubation time were optimized. Figure 7 shows the determination of illumination light intensity generated by LED. The volume of reaction mixture with a series of sample concentrations was fixed and illumination light intensity was varied to investigate the response of Si photodiode. The illumination light intensity was adjusted by regulating the current applied to the LED. The magnitude of the current was directly proportional to the light intensity of the LED. For a mixture solution volume of 6 μL, 3 light intensities were applied; the output of the photodiode is shown in Figure 7a. It was found that the signal of Si photodiode was proportional to the input light intensity and a wide linear range was obtained when the applied current was 14 μA. Too low and too high light intensity produced poor resolution. Therefore, it was determined that for a mixture volume of 6 μL, the LED intensity under 14 μA was appropriate. Similarly, a relationship of the mixture volume and the individually optimized LED intensities were investigated which is shown in Figure 7b.

Once the LED light intensity was determined, incubation time was studied. Since the sample and reagent volume were reduced significantly compared to the 100 μL applied by the associated commercial kit, the time could be reduced accordingly. An example is given in Figure 4a, a reaction mixture volume of 10 μL with the βHBA concentration at 0.5 mM was tested. A current of 33 μA was applied to the LED for determining the optimized light intensity for the working reagent volume (Figure 7b). The output of Si photodiode was recorded every minute to investigate the reaction rate of the enzyme reaction. The readout of the photodiode was monitored until no further variation with time was noted. The time point at that moment was then considered as the saturation time based on the enzyme kinetics for the enzyme reaction under the associated volume. Seven minutes, three minutes and one minute time points were investigated as the saturation time for the volume of 15 μL, 10 μL and 6 μL, respectively, as shown in Figure 4b.

After determination of the optimized illumination light intensity and the incubation time, standard curves for three representative volumes, 15 μL, 10 μL and 6 μL, were built (Figure 5). A series of βHBA standard solutions with the concentration ranging from 0.2 mM to 1.0 mM were measured by the presented biosensor. An overall lower photodiode signal was investigated with the change in the volume of the reagent mixture. For example, at the concentration of 0.5 mM, the outputs of the photodiode were 1.5 V, 2.3 V and 3.0 V for the volume of 15 μL, 10 μL and 6 μL, respectively. This result can be explained due to the linear relationship between the light absorption and the amount of absorbing substance.

### Optimization of operating parameters based on chip-based tests

The feasibility and detection performance of the microfluidic biosensor has been proved based on the results obtained by the well-based tests. One microliter of βHBA standard solution and detector mixture was loaded into the inlets on the microfluidic chip, simultaneously. The actual detected volume of mixture sample solution in the sensing well was 0.2 μL. Similarly, the appropriate LED intensity was determined for this 0.2 μL volume. As shown in Figure 6a, a relative higher or lower light intensity would result in a poor resolution and lead to decrease in the sensing range of samples. A current of 14 μA was determined as the appropriate LED intensity for the on-chip tests. After 1 min incubation time, the βHBA standard solution with a series of concentrations ranging from 0.05 mM to 1.0 mM was tested on chip to create the calibration curve for the microfluidic biosensor. The standard curve (Figure 6b) of the output of the microfluidic biosensor had good linearity with the βHBA at concentrations of 0.05 mM to 1.0 mM. The regression model was determined to be *y* = -0.4858*x* + 4.467 with *R*^2^ = 0.991. The detection limit of the microfluidic biosensor was 0.05 mM. The detection limit and the upper range tested are more than sufficient to distinguish healthy from SCK cows, particularly with sample dilution.

### βHBA determination in serum sample

**Figure 8:**
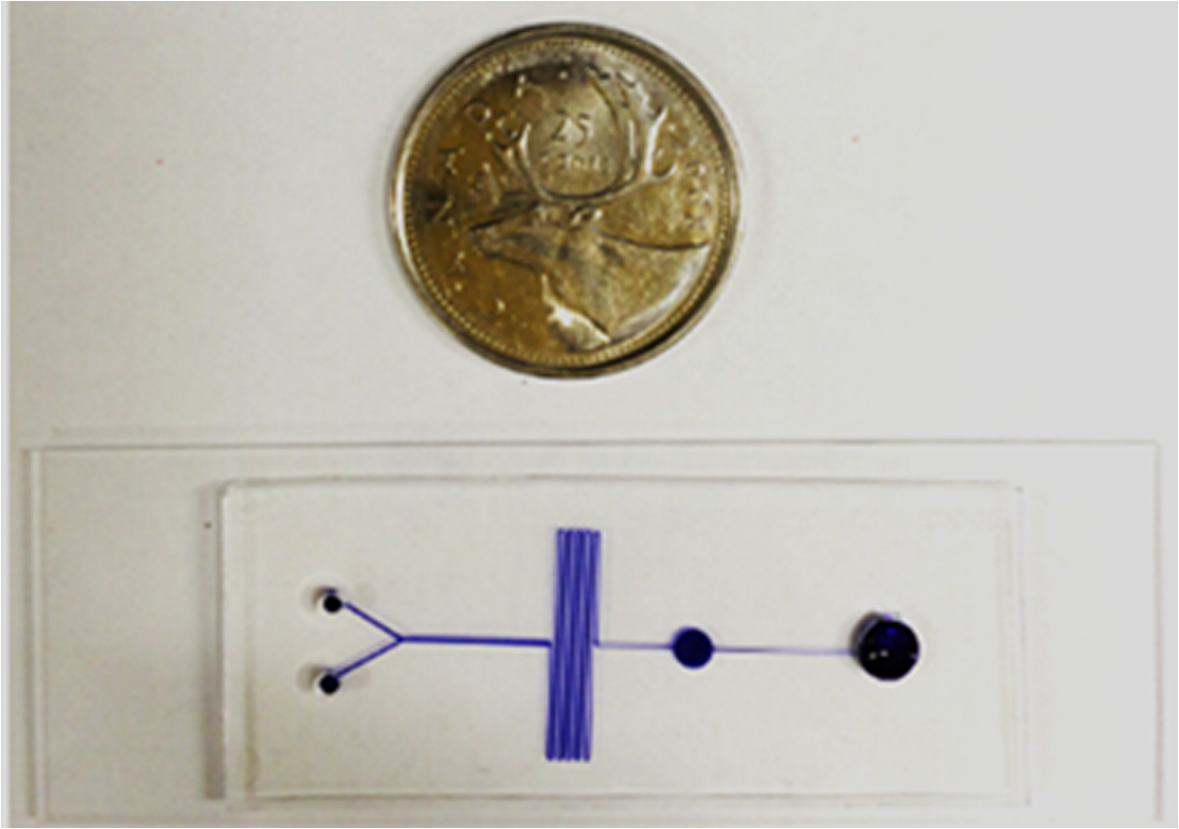
**Response of biosensor in determination of the βHBA concentration in serum samples.**

The detection performance of the microfluidic biosensor was compared to the other two laboratory methods, namely a chemistry analyzer and microplate reader. A series of serum samples and a control were assayed by the three methods on the same day. The serum samples were diluted to 1:6 ratio before conducting the assay experiments. The results of light signal captured by Si photodiode are shown in Figure 8. The values of the βHBA concentration in serum samples were then calculated using the obtained standard curve as shown in Figure 6b. The results of the detection of BHBA in blood samples using our developed microfluidic biosensor are in agreement with the microplate reader assay and the chemistry analyzer tests (Table 1). In addition, the microfluidic biosensor showed a good performance in assaying samples with βHBA concentration varying over a wide range, 0 mM to 5 mM, (Figure 6b) as the photodiode was able to sense higher concentration (>1.0 mM) with effective outputs (0 V to 3.9 V).

**Table 1:** **Comparison of βHBA determination in serum sample by three approaches**

**Serum sample #**	**Method**		
	**Chemistry analyzer [μM]**	**Microplate reader [μM]**	**Microfluidic biosensor [μM]**
1	627	530	580
2	1224	1277	1280
3	2399	2784	2433
4	3005	3093	3051
5	5152	4541	4985
Control	0	0.03	<0

Evaluation of human medicine hand-held devices [7,13,16] in the determination of βHBA in cow’s blood samples provided acceptable level of agreement with the laboratory tests but with significant false positive tests. Cows and humans are both mammals with considerable differences in their physiology. Cows have 11 major blood group systems (A, B, C, F, J, L, M, R, S, T and Z) unlike 4 groups in human system owing to different antigen expressions, which makes it complex and inaccurate to determine βHBA in cows using human medicine ketosis detector. Hence, human ketosis detector cannot be efficiently deployed for accurate and reliable measurement of βHBA in cow’s blood and animal samples.

Since the diagnosis and the determination of βHBA are dependent on a variety of body fluids, the hand-held commercially available human ketosis detectors are limited in its function. The uniqueness of the developed lab on a chip system is that the sensor has been built based on microfluidic systems with higher specificity and sensitivity along with higher accuracy for measurement in blood samples of cows. Unlike the human ketosis detection system, the developed microfluidic optical biosensing system is specific for determining βHBA in cow blood samples. The cross-reactivity detection between the target antigen and the analogues for other species is considered low due to the specificity and the sensitivity 0.95 nmol/mL sensitivity. The reagents and solutions used for the sensing event can be stored over 2 months upon refrigeration @ -80°C to avoid the loss of bioactivity and contamination.

## Conclusions

Conventional laboratory methods for βHBA determination are time-consuming, labour-intensive and requiring high sample/reagent consumption. Owing to the differences in the blood types and the antigen expression differences between the humans and cows, the commercially available human ketosis detection system cannot be efficiently employed for veterinary applications. A clear need exists in the miniaturization and simplification of the detection devices for βHBA. The developed microfluidic biosensor prototype overcomes the above limitations. Sample/ reagent consumption and incubation time were significantly reduced to 1 min and 0.2 μL respectively through microfluidic method. A miniaturized, low-cost optical sensor has been developed for the photometric detection and incorporated in the biosensor with 0.05 mM LOD. The presented microfluidic biosensor prototype in this study have its distinct advantages such as low cost, lower sample consumption and lower detection limit, which serves as an alternative for the development of rapid, accurate, highly sensitive and specific portable devices for on-farm and cowside diagnosis of SCK.
